# Influenza Virus Infection Induces a Narrow Antibody Response in Children but a Broad Recall Response in Adults

**DOI:** 10.1128/mBio.03243-19

**Published:** 2020-01-21

**Authors:** Philip Meade, Guillermina Kuan, Shirin Strohmeier, Hannah E. Maier, Fatima Amanat, Angel Balmaseda, Kimihito Ito, Ericka Kirkpatrick, Andres Javier, Lionel Gresh, Raffael Nachbagauer, Aubree Gordon, Florian Krammer

**Affiliations:** a Graduate School of Biomedical Sciences, Icahn School of Medicine at Mount Sinai, New York, New York, USA; b Department of Microbiology, Icahn School of Medicine at Mount Sinai, New York, New York, USA; c Centers of Excellence for Influenza Research and Surveillance (CEIRS); d Center for Research on Influenza Pathogenesis (CRIP), New York, New York, USA; e Sustainable Sciences Institute, Managua, Nicaragua; f Centro de Salud Sócrates Flores Vivas, Ministry of Health, Managua, Nicaragua; g Department of Epidemiology, School of Public Health, University of Michigan, Ann Arbor, Michigan, USA; h Laboratorio Nacional de Virología, Centro Nacional de Diagnóstico y Referencia, Ministry of Health, Managua, Nicaragua; i Division of Bioinformatics, Hokkaido University Research Center for Zoonosis Control, Kitaku, Japan; j St. Jude Center of Excellence for Influenza Research and Surveillance, Memphis, Tennessee, USA; St. Jude Children's Research Hospital

**Keywords:** influenza virus, natural infection, imprinting, heterosubtypic immunity, cross-reactivity, influenza

## Abstract

In contrast to influenza virus vaccination, natural infection induces long-lived and relatively broad immune responses. However, many aspects of the antibody response to natural infection are not well understood. Here, we assessed the immune response after H1N1 influenza virus infection in children and adults in a Nicaraguan household transmission study using an influenza virus protein microarray (IVPM). This technology allows us to simultaneously measure IgG and IgA antibody responses to hemagglutinins of many different virus strains and subtypes quantitatively with a high throughput. We found that children under 6 years of age responded to natural infection with a relatively narrow response that targeted mostly the hemagglutinin of the strain that caused the infection. Adults, however, have a much broader response, including a boost in antibodies to many group 1 subtype hemagglutinins. Also, a strong recall response against historic H1 hemagglutinins that share the K133 epitope with the pandemic H1N1 virus was observed. Of note, some children, while responding narrowly within H1 and group 1 hemagglutinins, induced a boost to H3 and other group 2 hemagglutinins when infected with H1N1 when they had experienced an H3N2 infection earlier in life. This is an interesting phenomenon providing evidence for immune imprinting and a significant new insight which might be leveraged in future universal influenza virus vaccine strategies. Finally, preexisting immunity to pandemic H1 hemagglutinins was significantly associated with protection from infection in both children and adults. In adults, preexisting immunity to non-H1 group 1 hemagglutinins was also significantly associated with protection from infection.

## Abstract (precis)

It is known since Thomas Francis, Jr. published his first paper on original antigenic sin in 1960 that the first infection(s) with influenza virus leaves a special immunological imprint which shapes immune responses to future infections with antigenically related influenza virus strains. Imprinting has been implicated in both protective effects as well as blunting of the immune response to vaccines. Despite the fact that this phenomenon was already described almost 60 years ago, we have very little detailed knowledge of the characteristics and breadth of the immune response to the first exposure(s) to influenza virus in life and how this compares to later exposure as adults. Here, we investigate these immune responses in detail using an influenza virus protein microarray. While our findings are mostly descriptive in nature and based on a small sample size, they provide a strong basis for future large-scale studies to better understand imprinting effects.

## INTRODUCTION

**FIG 1:**
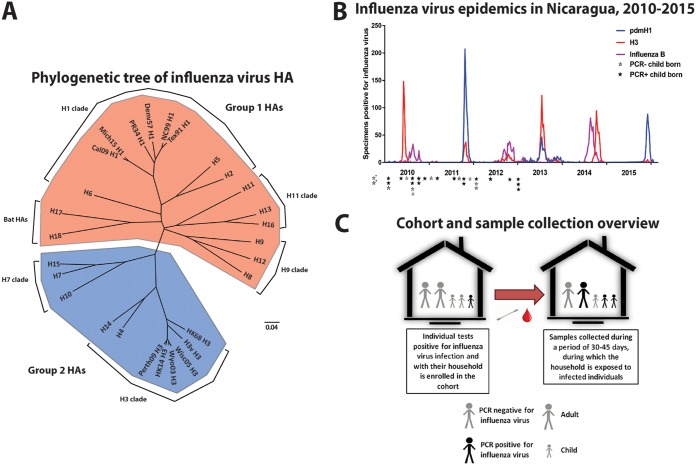
Study design and overview. (A) Phylogenetic tree showing all group 1 and group 2 HA subtypes and including all strains used in this analysis. The tree is based on amino acid sequences and was built in Clustal Omega and visualized with FigTree. The scale bar represents a 4% difference in amino acid composition. (B) Circulation of H1N1, H3N2, and influenza B viruses in Nicaragua between 2010 and the beginning of 2016. The graph is based on data sourced from the World Health Organization. Samples analyzed were taken during the 2015–2016 season. Black stars represent the birthdates of children who turned PCR^+^ during the 2015–2016 season, gray stars indicate the birthdates of children who stayed PCR^−^, and asterisks denote children who were born before circulation data were recorded. (C) Overall design of the transmission study. Index cases were identified, and their families were enrolled. Blood samples were collected at enrollment (pre samples). Households were followed with intensive monitoring periods of 10 to 14 days, with nasal swabs collected every 2 to 3 days. A follow-up blood sample of all family members was performed at 30 to 45 days after enrollment (post samples).

Influenza virus infections are a major global public health problem. Current vaccines work when well matched to circulating pathogenic strains but induce narrow and often short-lived antibody responses (1). In contrast, it has been shown that natural infection can induce long-lived (potentially lifelong) and broader immune responses (1). However, many aspects of the humoral immune response to natural infection are still not well understood. Typically, hemagglutination inhibition (HI) titers against a small panel of viruses of the same subtype that caused the infection are assessed to define immune responses, but little attention is given to hemagglutinin (HA)-binding antibodies. The true breadth of the immune response induced by natural infection with respect to antibodies binding to historic strains and heterosubtypic hemagglutinins (HAs), including group 1 (H1, H2, H5, H6, H8, H9, H11, H12, H13, H16, H17, and H18) and group 2 (H3, H4, H7, H10, H14, and H15) HAs is unknown (Fig. 1A). However, it has recently been shown that HA-binding antibodies are an independent correlate of protection (2). Of note, many antibodies with antiviral functions, including those against the conserved stalk domain of HA, are not detected using traditional assays (1). Another important aspect is how the exposure history to influenza viruses shapes the breadth of the immune response to infection. The first exposure(s) to influenza virus in life leaves an immunological imprint in humans, historically referred to as original antigenic sin (3). This imprinting has recently been shown to play major roles in shaping the antibody response after both natural infection as well as vaccination (4–11). In this respect, it is also unclear if children (who have very little immune history but might already be imprinted by their first infection) have a different response and breadth of response to infection from those of adults (who have extensive preexposure histories to influenza virus, including imprinting events early in their lives). In the past, we have used enzyme-linked immunosorbent assays (ELISAs) and generated antigenic landscapes to explore these questions (12). However, performing individual ELISAs against recombinant HAs of many different virus strains and subtypes is tedious and time-consuming and can be sample intensive. Recently, we therefore developed influenza virus protein microarrays (IVPMs) (13). For this technology, we print a library of recombinant HA proteins, including all HA subtypes, on microarrays which are then probed with serial dilutions of serum (see Fig. S1 in the supplemental material). This allows us to quantitatively assess the breadth of the antibody response to a large number of HAs. Here, we used this technology to investigate the immune response in a household influenza transmission study in Nicaragua. Pre- and postexposure samples of children and adults infected (PCR^+^) with pandemic H1N1 virus were characterized alongside exposed but noninfected (PCR^−^) children and adults to learn more about the breadth of immune responses to natural influenza virus infection.

10.1128/mBio.03243-19.1 FIG S1 Schematic of the influenza virus protein microarray pipeline. (A and B) Proteins are expressed in a baculovirus expression system, purified on Ni-nitrilotriacetic acid resin columns (A) and spotted on epoxysilane-coated glass slides (B). (C) The slides are then probed with serum dilutions and fluorescently labeled secondary antibodies in gaskets that house 4 glass slides per gasket. Each slide is further subdivided into 24 wells by the gaskets, in which serial dilutions are performed. (D and E) Slides are read on a microarray plate reader (D), and the data are analyzed (E). Download FIG S1, DOCX file, 1.2 MB. Copyright © 2020 Meade et al. 2020 Meade et al. This content is distributed under the terms of the Creative Commons Attribution 4.0 International license.

## RESULTS

### Study design.

In this study, we tested sera from a Nicaraguan household influenza transmission study that were collected during November and December 2015 (Fig. 1B). Briefly, influenza index cases were identified and their households enrolled (Fig. 1C) (14). Blood samples were collected from all household members at enrollment. Households were then intensively monitored for 10 to 14 days, during which time nasal/oropharyngeal swabs were collected every 2 to 3 days to be tested by reverse transcription-PCR (RT-PCR) for virus, according to CDC protocols (15). Follow-up blood samples were collected 30 to 45 days after enrollment. While index cases might have already had clinical signs and virus replication, they presented to the study clinic within 1 day on average of reported symptom onset, and it was likely before the onset of the plasmablast response (which typically peaks at day 7 postinfection) (16, 17). Therefore, these subjects likely had titers close to baseline; samples for this time point are designated “pre,” while titers collected after the observation period are designated “post.” The ongoing epidemic in Nicaragua at this time was caused by a pandemic H1N1-like virus, with very few H3N2 infections reported nationwide (Fig. 1B). Subjects were grouped into children (<6 years of age) and adults (18 to 49 years of age) and into PCR^+^ and PCR^−^. Since we wanted to study differences between young children and adults, 7- to 17-year-old individuals were excluded. The tested groups were children with PCR-confirmed influenza virus infection (PCR^+^ children, *n* = 18), children with negative PCR and no illness (PCR^−^ children, *n* = 11), adults with PCR confirmed influenza virus infection (PCR^+^ adults, *n* = 19), and adults with negative PCR and no illness (PCR^−^ adults, *n* = 17). Of note, all children were old enough to have experienced several influenza epidemics in Nicaragua (Fig. 1B). In order to assess the antibody breadth at baseline and that induced by infection, we used an IVPM that featured at least one recombinant HA from each subtype and from several strains each for H1 and H3 (Fig. 1A). For H1, the strains chosen included prepandemic seasonal strains A/Puerto Rico/8/34 (PR34 H1), A/Denver/1/57 (Denv57 H1), A/Texas/36/91 (Tex91 H1), and A/New Caledonia/20/99 (NC99 H1) and pandemic-like A/California/04/09 (Cal09 H1) and A/Michigan/45/15 (Mich15 H1) viruses. Of note, only Tex91 H1, Cal09 H1, and Mich15 H1 contained the K133 epitope (10), and Mich15 H1 was antigenically closest to the strains circulating in Nicaragua in 2015. Recombinant HAs for H3 strains included A/Hong Kong/1/68 (HK68 H3), A/Wyoming/03/03 (Wyo03 H3), A/Wisconsin/67/05 (Wisc05 H3), A/Perth/16/09 (Perth09 H3), and A/Hong Kong/4801/14 (HK14 H3), as well as the H3 variant strain A/Indiana/10/11 (H3v). These HAs were printed on epoxysilane glass microarray slides and probed with sera, and then IgG and IgA signals were read in parallel from the same arrays using two differently labeled secondary antibodies (Fig. S1). Protein spots were printed in triplicate for each array, and samples were assayed in three different dilutions. The reported data are based on the area under the curve (AUC) calculated from the average signal for each protein at each dilution. This method was chosen because it is more quantitative than assaying one serum dilution only. We have also shown in the past (13) and here that the method correlates well with ELISA results, can detect antibodies with high specificity, and can detect broadly neutralizing antibodies that bind to fragile and conformational epitopes (Fig. S2).

10.1128/mBio.03243-19.2 FIG S2 Titers measured with the IVPM technology correlate with ELISA titers. (A) In a pilot study, seven commercially obtained human serum samples were probed on the IVPM against five different recombinant HAs. The data are represented as a heat map of AUC values. (B) Same serum/protein combinations but measured by ELISA. (C) Correlation between IVPM and ELISA data. (D) MAb CR9114, which binds to all influenza A virus HA subtypes and influenza B virus HA in ELISA (28), also binds broadly to different HAs on the IVPM. (E) To demonstrate specificity, the IVPM was also probed with pandemic H1 specific (as assessed in ELISA) MAb 4C04 (37). The MAbs show the same specific pattern of reactivity on an IVPM as assessed in ELISA. Download FIG S2, DOCX file, 0.3 MB. Copyright © 2020 Meade et al. 2020 Meade et al. This content is distributed under the terms of the Creative Commons Attribution 4.0 International license.

### Adults and children differ in IgG reactivity landscapes after H1N1 infection.

**FIG 2:**
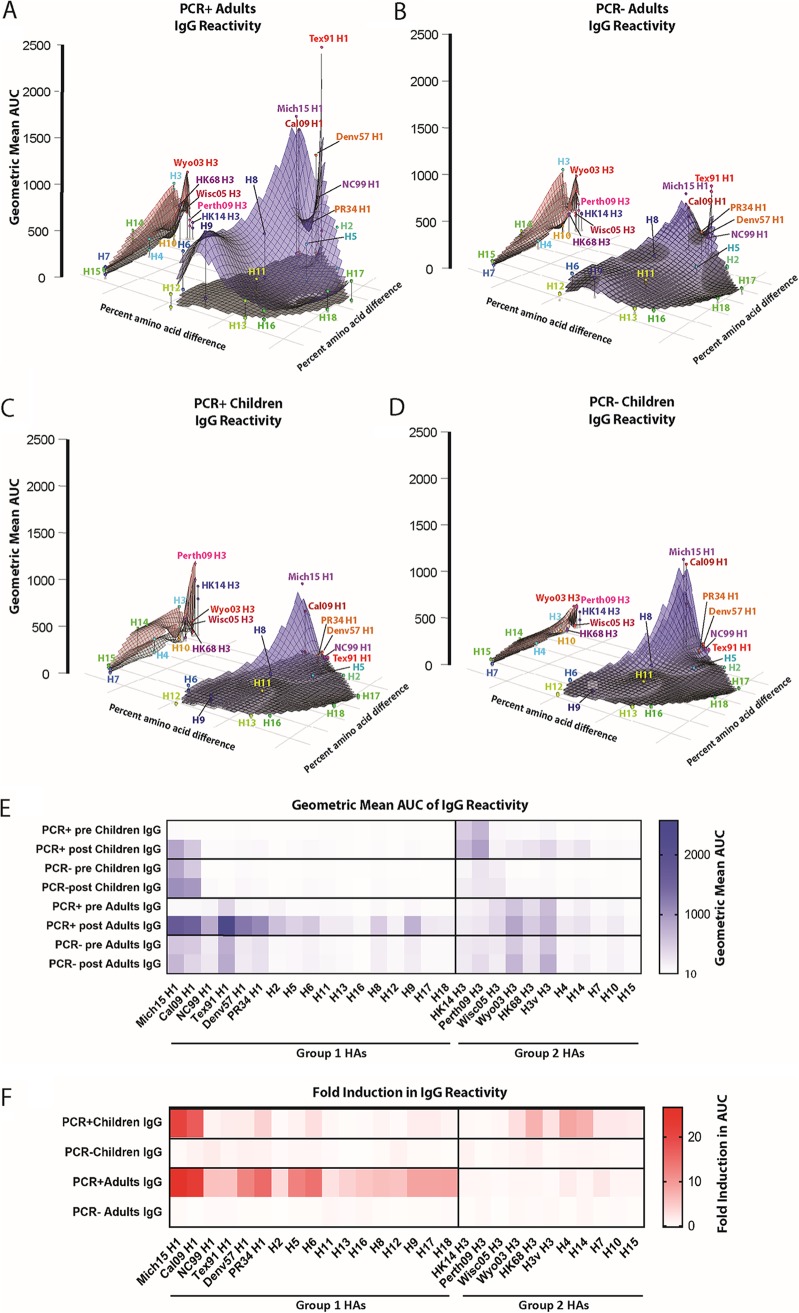
IgG antigenic landscapes of adults and children pre- and postexposure. Influenza virus protein microarray (IVPM) AUC values and amino acid sequences were used to generate antigenic landscapes using multidimensional scaling. The *z* axis represents reactivity to a substrate (AUC as geometric mean titer), and the *x* and *y* axes represent amino acid differences between HAs used as the substrate. The gray plane under each of the red and blue planes represents the preexposure reactivity, the blue plane represents the postexposure group 1 reactivity, and the red plane represents the postexposure group 2 reactivity. The different strains/subtypes are indicated by colored spheres labeled with the substrate name. (A) PCR^+^ adults. (B) PCR^−^ adults. (C) PCR^+^ children. (D) PCR^−^ children. (E and F) Shown are the same data in a heat map and the fold induction in a heat map format (F) (as geometric mean induction).

To visualize the breadth of reactivity toward different HAs before and after H1N1 infection, we used multidimensional scaling (MDS) to generate antigenic landscapes (Fig. 2A to D). AUC values for each HA were plotted onto an inferred HA landscape that is based on the amino acid sequences of the HAs used. This method of visualization allows for easy identification of differences between pre and post sera and between groups. PCR^+^ adults had relatively low baseline IgG titers against group 1 HAs, with a small peak toward Tex91 H1 HA, while PCR^−^ adults showed higher baseline values to group 1 HAs with peaks for both prepandemic seasonal and pandemic H1 HAs (Fig. 2A and B). The H3 IgG baseline values for both groups were similar and focused on moderately recent H3 HAs (e.g., Wyo03 H3) with some cross-reactivity to other group 2 HAs. Post sera for PCR^+^ adults showed a strong induction of IgG antibodies across group 1 HAs with peaks for pandemic H1 HAs and prepandemic seasonal H1 HAs (Fig. 2A). On average, negligible induction for group 2 HAs was observed. No IgG induction was observed in post sera from PCR^−^ adults (Fig. 2B). In PCR^+^ children, the baseline IgG levels were low against group 1 HAs in general, with low reactivity to pandemic H1 HAs, while most of them had preexisting immunity to recent H3 strains (e.g., HK14 H3) but not to other group 2 HAs (Fig. 2C). PCR^−^ children already had higher IgG titers against pandemic H1 HAs but not against prepandemic seasonal or other group 1 HAs (Fig. 2D). In addition, the PCR^−^ children had slightly lower baseline IgG titers against group 2/H3 HAs than did the PCR^+^ children. While adults mounted a broad group 1 IgG response after H1N1 infection, PCR^+^ children mounted a surprisingly narrow response that was limited to the two pandemic H1 HAs that were antigenically closely related to the pandemic H1N1 (H1N1pdm) viruses with which the children were infected (Fig. 2A and C). Very little change was observed in the PCR^−^ group of children. Notably, overall, PCR^+^ children also induced IgG antibodies to group 2 HAs, including moderately strong responses to H3, H4, and H14 (all H3 clade). Since it can be hard to distinguish specific strains in MDS landscapes, we also visualized the AUC values using heat maps (Fig. 2E). This visualization, and the calculated fold induction (Fig. 2F), reflect the observations from the MDS plots. In summary, adults mounted broad IgG antibody responses to group 1 HAs after pandemic H1N1 infection, while children produced relatively strain-specific antibody responses to pandemic H1N1 HAs. Surprisingly, some children also mounted a broad group 2 IgG response.

### IgA reactivity is low pre- and postinfection but correlates with IgG titers.

**FIG 3:**
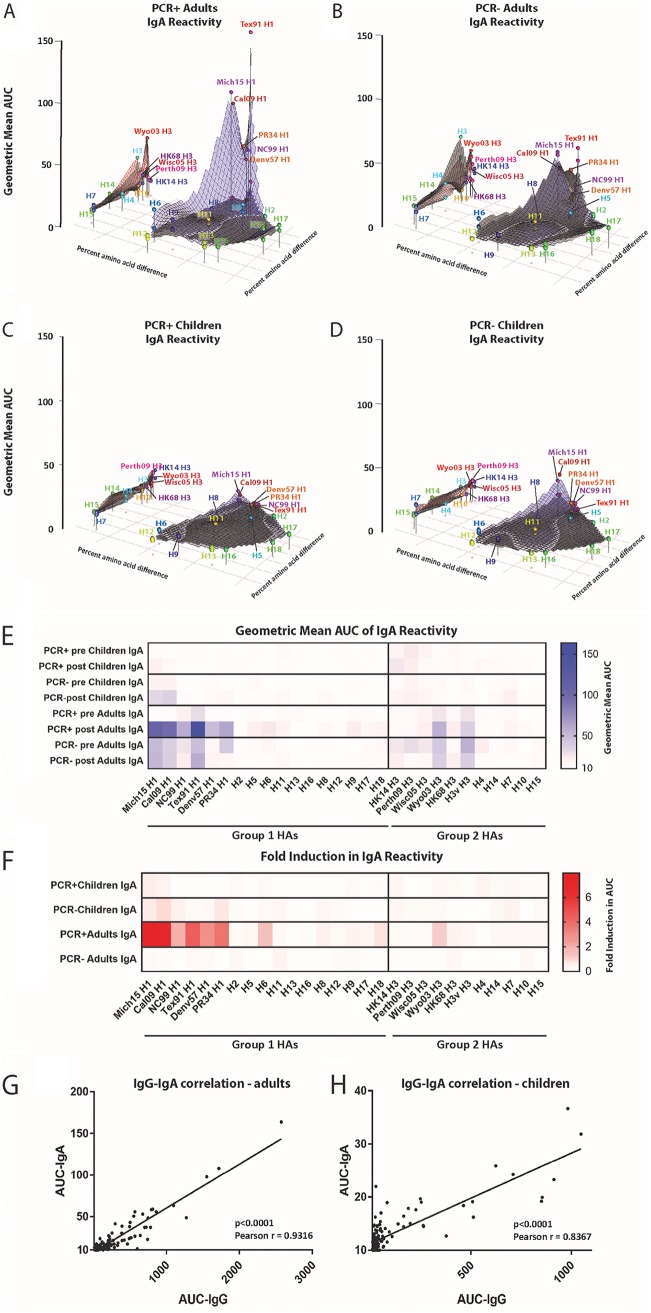
IgA antigenic landscapes of adults and children pre- and postexposure. Influenza virus protein microarray (IVPM) AUC values and amino acid sequences were used to generate antigenic landscapes using multidimensional scaling. The *z* axis represents reactivity to a substrate (AUC as geometric mean titer), and the *x* and *y* axes represent amino acid differences between HAs used as the substrate. The gray plane under each of the red and blue planes represents the preexposure reactivity, the blue plane represents the postexposure group 1 reactivity, and the red plane represents the postexposure group 2 reactivity. The different strains/subtypes are indicated by colored spheres labeled with the substrate name. (A) PCR^+^ adults. (B) PCR^−^ adults. (C) PCR^+^ children. (D) PCR^−^ children. (E and F) Shown are the same data in a heat map and the fold induction in a heat map format (F). (G and H) Correlation analysis between IgG and IgA titers for adults (G) and children (H).

Next, using MDS plots again, we assessed IgA reactivity. This is interesting and important since it has been proposed that the IgA repertoire might be more cross-reactive than the IgG repertoire (18). As expected, we found that IgA titers were in general lower than IgG titers (Fig. 3). In adults, IgA findings were mostly reflective of the IgG findings. PCR^+^ adults had low baseline values against both group 1 and 2 HAs, with moderate peaks for Tex91 H1 and Wyo03 H3. PCR^−^ adults had similar reactivity but higher titers against pandemic H1N1 HAs (Fig. 3A and B). Post reactivity in PCR^+^ adults increased against pandemic and seasonal H1 HAs, while no change was seen for PCR^−^ adults. Interestingly, the breadth of the IgA response in PCR^+^ adults was lower than that for IgG, with relatively little induction of group 1 HA titers detected. IgA titers in pre and post sera from PCR^+^ children were negligible, and PCR^−^ children had slightly elevated IgA titers against H1 in the pre sera that did not change over time (Fig. 3C and D). Again, to make reactivity to specific strains more visible, we also visualized the data as a heat map (Fig. 3E), with induction shown in Fig. 3F. We further performed a correlation analysis for IgG versus IgA in adults and children and found low but significant correlation in both cases (Fig. 3G and H). In summary, IgA titers were lower in all four groups and less broad than the IgG titers in adults but showed moderate correlation with IgG titers in adults and children.

### H3N2 preexposed children mount a response to pandemic H1 and group 2 HAs after H1N1 infection.

**FIG 4:**
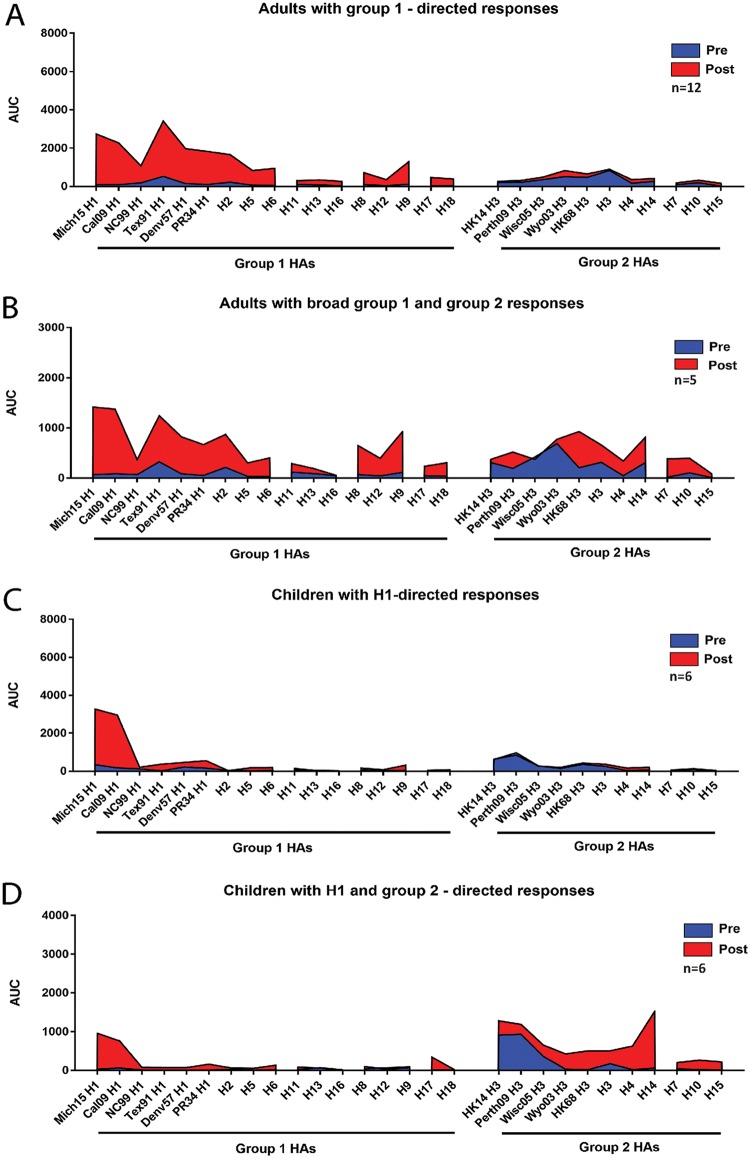
IgG reactivity profiles. PCR^+^ adults and children were binned into categories each based on their reactivity profile. The *y* axis of these plots shows the geometric mean AUC of the group, and the different HAs are plotted on the *x* axis. (A) Adults who induce a predominant group 1 response. (B) Profile of adults who induce IgG against both group 1 and group 2 HAs. (C) Reactivity profile of children who mount a narrow pandemic H1 HA response. (D) Reactivity of children who mount a narrow pandemic H1 HA response plus a response to group 2 HAs.

To better understand the responses mounted by PCR^+^ adults and children, we compared the reactivity profiles of individuals and grouped them into four categories (Fig. 4). The majority of the PCR^+^ adults (*n* = 12) showed an induction to H1 HAs related to the strain causing the infection, to prepandemic seasonal H1 HAs, as well as to other group 1 HAs (Fig. 4A). Specifically, antibody titers to other members of the H1 clade (H2, H5, and H6) and the H9 clade (H8, H9, and H12) were boosted, with less induction of reactivity to the H11 clade (H11, H13, and H16) and the bat HAs (H17 and H18). In this group of individuals, no induction of cross-group antibodies (in this case to group 2 HAs) was observed. A minority of PCR^+^ adults also showed induction of cross-group antibodies after pandemic H1N1 infection in addition to the broad group 1 induction (Fig. 4B). Curiously, this group 2 induction was not necessarily focused on recent H3 strains but on HAs from older H3 strains, as well as other subtypes from the H3 (H3, H4, and H14) and H7 (H7, H10, and H15) clades. The majority of PCR^+^ children had a narrow response specific to pandemic H1 HA with negligible cross-reactivity to other H1 HAs or group 1 HAs. Six of these PCR^+^ children (33%) had a very narrow response to the pandemic H1 HAs, with slightly higher titers to the better-matched Mich15 H1 HA than to Cal09 H1 HA (Fig. 4C). Another six PCR^+^ children (33%) showed a very distinct response. In addition to the narrow response to the pandemic H1 HAs, they mounted a strong response to group 2 HAs with a preference to older H3 strains, H4 and H14 (Fig. 4D). In this group, in comparison to the pandemic H1 HA-only-reacting group of PCR^+^ children, the preexisting baseline reactivity to H3 was higher, which might have played a role in this peculiar response. The remaining children were low H1 responders (*n* = 3), had a low response to H1 but responded well to group 2 (*n* = 2), or responded broadly to H1 and group 2 (*n* = 1).

### A recall response against the H1 K133 epitopes is dominant in adults.

**FIG 5:**
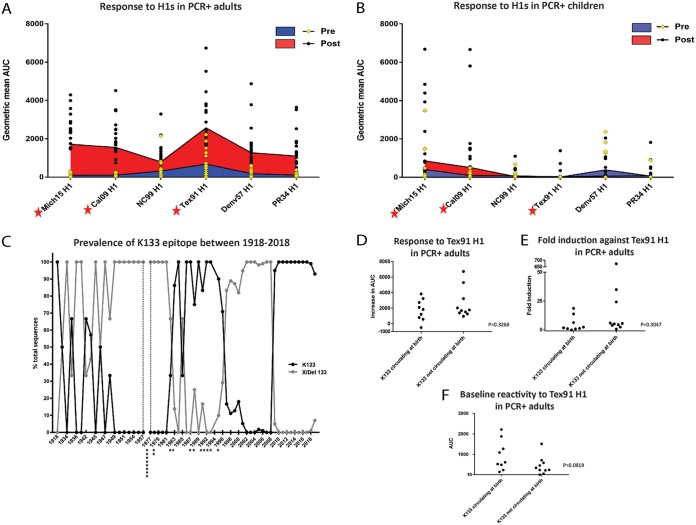
Back-boosting to an HA carrying the K133 epitope. (A and B) Reactivity of PCR^+^ individuals to different H1 HAs for adults (A) and children (B) (yellow diamonds indicate preexposure; black circles indicate postexposure; red stars indicate HAs that carry the K133 epitope). Shaded areas represent the geometric mean AUC of the group, with baseline reactivity (pre) in blue and postexposure (post) reactivity in red. (C) Frequency of the K133 epitope in H1N1 isolates over time. The black line represents K133, and the gray line represents viruses with other amino acids at position 133 or a deletion of the locus. The black stars indicate the number of PCR^+^ adults with particular birthdates. PCR^+^ adults were then grouped into a cohort born when viruses with K133 were the dominant circulating strain (1983 to 1996) and into a cohort which was exposed early in life to a non-K133 virus (before 1983). (D to F) Absolute differences between preexposure and postexposure, the fold induction, and the absolute pretiters for these two groups.

As described above, pandemic H1N1 infection caused an induction of antibodies to prepandemic seasonal H1 HAs in adults but not in children. Responses to one H1 HA, Tex91 H1, stood out specifically, as they were often higher than the responses to the HA of the infecting virus (Fig. 5A and B). It has been described that pandemic H1N1 infection induces antibodies that target the K133 epitope but only for age groups that were imprinted with seasonal H1N1 viruses carrying K133 as well (mainly 1983 to 1996; Fig. 5C) (10). Besides the two pandemic H1N1 HAs (Cal09 H1 and Mich15 H1), only Tex91 H1 on the IVPM array carries the K133 amino acid, which is absent (the whole position is deleted) from the other tested H1 HAs. When we had a closer look at H1N1 reactivity of all PCR^+^ adults, we clearly saw that the highest reactivity in pre- and postinfection sera was toward Tex91 H1 for a majority of individuals, and the induction for this H1 was higher than for all other prepandemic seasonal H1 HAs, suggesting a clear back-boost (Fig. 5A). This was not observed for PCR^+^ children (Fig. 5B). We therefore mapped the birth years of the PCR^+^ adults on a timeline that represents the frequency of the K133 epitope between 1918 and 2018 (Fig. 5C). We then divided the PCR^+^ adults into a group that likely was imprinted with a K133 epitope bearing H1N1 (1983 to 1996) and compared them to the group that was likely imprinted by a virus in which the K133 epitope was absent (born before 1983). Typically, the first exposure to influenza virus happens in the first few years of life. Given an attack rate of approximately 23% in unvaccinated children (19), it is very likely that the first exposure happens close to birth. We found no significant differences in change of reactivity (Fig. 5D), fold induction (Fig. 5E), or baseline reactivity (Fig. 5F) to Tex91 between the two groups, although there was a trend toward higher baseline reactivity in adults who were likely imprinted with a K133 virus.

### Higher preexisting reactivity to group 1 HA, the H1 subtype, and pandemic H1 HA correlates with protection.

**FIG 6:**
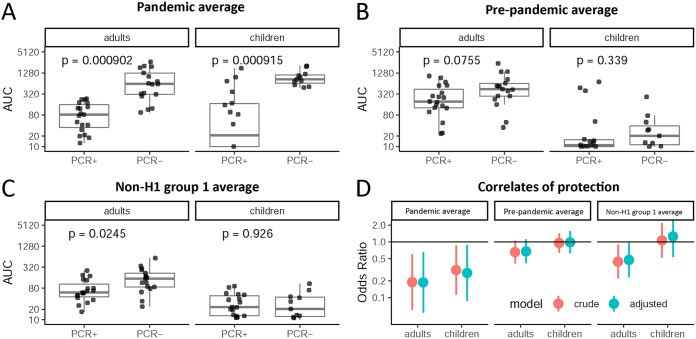
Preexisting group 1 anti-HA titers by infection status and correlates of protection. (A to C) Mean preexisting anti-HA titers (AUC) by PCR status and age for the 2 pandemic H1 strains (A), all prepandemic seasonal H1 strains (B), and all non-H1 group 1 strains (C). (D) Odds ratios for infection based on preexposure titers against pandemic H1 HA, prepandemic seasonal H1 HAs, and non-H1 group 1 HAs for adults and children. Unadjusted and models adjusted for age and sex are presented. Odds ratios are for a 2-fold increase in titer.

Finally, we wanted to assess the relationship between preexisting HA antibody levels and the risk of pandemic H1N1 infection. First, we plotted the averaged preexisting antibody AUCs for pandemic H1 HAs, prepandemic seasonal H1 HAs, and non-H1 group 1 HAs for PCR^+^ and PCR^−^ individuals. Especially the non-H1 group 1 titers are a representation of the “antigenic altitude” of a subject in the MDS plots. This analysis was performed for adults and children separately. For both adults and children, preexisting antibody levels to pandemic H1 HA were higher in the PCR^−^ groups (Fig. 6A). For adults, the same applied to reactivity to prepandemic H1 HA, where PCR^−^ individuals had higher titers than did PCR^+^ individuals (Fig. 6B). This was also the case in children but at lower reactivity in general (Fig. 6B) and with 3 children actually showing high reactivity in the PCR^+^ group. For adults, preexisting antibodies to non-H1 group 1 HAs were higher in PCR^−^ individuals (Fig. 6C), again suggesting the greater breadth of response in adults. This was not the case in children, who had lower preexisting antibodies to non-H1 group 1 HAs in general (Fig. 6C). Next, we examined protective effects associated with a 2-fold increase in antibody levels adjusted for age and sex. We found that in adults, preexisting antibodies to pandemic H1 HAs (odds ratio [OR], 0.19; confidence interval [CI], 0.06 to 0.66) and non-H1 group 1 HAs (OR, 0.48; CI, 0.23 to 0.98) were significantly associated with protection from PCR-confirmed infection (Fig. 6D). In children, preexisting antibodies to pandemic H1 HAs were significantly associated with protection (OR, 0.28; CI, 0.09 to 0.88), but antibodies to non-H1 group 1 HAs were not (Fig. 6D). In both children and adults, antibodies to prepandemic H1s were not associated with protection (Fig. 6D).

## DISCUSSION

Using the IVPM technology, we have made several interesting observations in this study. The most intriguing finding is the difference in the breadth of the antibody responses in children and adults. Children showed a very narrow antibody response after infection that, for group 1 HAs, targeted only pandemic H1N1-like HAs, which are antigenically related to the virus causing the infection. In contrast, adults experienced a back-boost to a broad range of seasonal H1 and other group 1 HAs. This observation suggests that the initial exposure of children to influenza viruses causes a biased response to immunodominant and strain-specific epitopes, a phenomenon that can also be observed in naive mice (12, 20). It further suggests that levels of cross-reactive antibodies in adults increase over time and that infection with a group 1 HA-expressing virus, like pandemic H1N1, induces broader responses to divergent H1 HAs as well as other group 1 HAs in an original antigenic sin-like fashion (3). This response might include higher titers of antibodies to the stalk domain, which have been shown to be elevated in older individuals when probed cross-sectionally or longitudinally (21, 22). This increase in cross-reactive anti-stalk antibodies with age has been attributed to sequential exposure to divergent group 1 HAs (H2, prepandemic seasonal H1, and pandemic H1) that share a conserved stalk but divergent head domains (1). Of note, titers are much lower for group 2 stalk antibodies in adults and the elderly, likely because population-level exposure to group 2 HA-expressing viruses has been limited to seasonal H3N2 (21, 22). Our data are corroborated by several other studies that report narrow immune responses in children in response to natural infection or vaccination (23, 24). In addition, it has been shown on a monoclonal and polyclonal level that older individuals have broader baseline antibody reactivity (21) and might mount broader antibody responses after exposure to influenza virus antigens (22, 25–27).

Another phenomenon that was observed was strong reactivity toward Tex91 in adults after exposure to pandemic H1N1. Tex91 and pandemic H1N1 HAs share a conserved epitope in the head domain centered around K133 (10). It has been shown previously that individuals born when a K133-carrying virus was circulating mounted a K133-focused response to the pandemic H1N1 HA (10). Individuals that were born when non-K133-expressing viruses circulated did not show this focused immune response to the K133 epitope of pandemic H1N1 (10). These differences were not observed in our study, as K133- and non-K133-imprinted adults had a similar back-boost to Tex91. However, baseline titers to Tex91 were higher in K133-imprinted individuals. Differences between this and other studies might be caused by the different assays and methodologies used. As an alternative explanation, the number of subjects might have been too small to detect differences between the groups. However, the magnitude of baseline cross-reactivity to Tex91 and of the boost after pandemic H1N1 exposure validates the importance of this epitope for immunity to H1N1 viruses.

As described above, children mounted a very narrow response to the H1 HAs closely related to the infecting strain and did not induce cross-reactive antibodies to other H1 or group 1 HAs. However, a third of the children also mounted a response toward group 2 HAs, especially older H3 HAs, H4, and H14. Of note, this group of children had been preexposed to H3N2 and had higher titers to recent H3 HAs than those of the group that mounted an HA response specific to pandemic H1N1. It is unclear what caused this strong cross-reactivity. Initially, we did consider that coinfections with H3N2 could have occurred. However, circulation of H3N2 in the analyzed season in Nicaragua was negligible; therefore, this scenario is unlikely to have occurred in a third of the H1N1-infected children. Another scenario could be that these children came in contact with avian influenza viruses, e.g., H4 or H14, which could have caused this induction or which could have caused an imprinting pattern that then triggered this peculiar response after pandemic H1N1 infection. However, it is unlikely that this occurred in 33% of PCR^+^ children. Another possibility is that a strong H3N2 priming through natural infection can leave an imprint that also influences the response to group 1 viruses such as pandemic H1N1. It is possible that this response is driven by anti-stalk antibodies since cross-group-reactive antibodies have been reported (28–30). Most cross-group stalk-reactive antibodies would likely target all group 1 and group 2 HAs and not specifically pandemic H1 plus group 2 HAs (28–30). However, recently, three studies reported VH3-53 germ line anti-stalk antibodies after H7N9 vaccination that showed the same pattern of targeting pandemic H1N1 plus broad group 2 binding (31–33). Another class of antibodies that could be involved in this phenomenon are antibodies that bind the head trimer interface and have recently been reported in humans as well (34).

The observed phenomena including the back-boost in adults against group 1 HAs and against the K133 epitope as well as the H1-group 2 cross-reactivity in children are evidence for the complex response of the immune system to (sequential) exposure to influenza viruses (5, 6, 9). Our findings certainly warrant follow-up studies with larger numbers of subjects and longitudinal analyses to shed more light on mechanisms behind back-boosting and imprinting effects. Analysis of the antibody response on a monoclonal antibody level would also help assess which specific class of antibody is responsible for the observed cross-reactivity.

Another interesting observation was the difference between IgG and IgA serum responses. The IgG response was higher in signal strength. While this could be influenced by the different secondary antibodies used in the analysis, it is likely also a reflection of the larger amount of IgG than IgA in serum. Surprisingly, the IgG response in adults was broader than the IgA response, which is in contrast to assumptions made based on human B-cell analysis (18). Furthermore, the IgA response to infection was relatively low in children, which is unexpected since antigen presented on mucosal surfaces is expected to drive stronger IgA responses (which might still be the case for local mucosal immunity, which was not assessed in our study). However, IgG and IgA responses still correlated moderately in both children and adults.

Finally, we analyzed if preexisting immunity would be predictive of the risk of getting infected with pandemic H1N1. In adults, preexisting antibody titers to pandemic H1 HAs and non-H1 group 1 HAs were associated with significant protection from infection; antibodies to prepandemic H1 strains were somewhat higher among PCR^−^ adults but not associated with significant protection. In children, only preexisting antibodies to the pandemic H1 HAs were associated with significant protection from infection. However, it needs to be kept in mind that children had very low levels of antibody to seasonal H1 and non-H1 group 1 HAs. This finding indicates that binding antibodies as measured in the IVPM might serve as correlate of protection. We have also shown this recently with ELISA data against a specific H1 HA and the stalk of H1 (2).

The small number of subjects tested and the relatively low number of HAs probed do not allow us to draw firm conclusions. However, this study serves to generate hypotheses regarding imprinting and the evolution of antibody responses during sequential exposure to natural infection with influenza viruses. In conclusion, we show that children mount a much narrower antibody response to pandemic H1N1 infection than do adults, who respond broadly to group 1 HAs. Notably, a subpopulation of children induces pandemic H1 HA plus group 2 HA reactivity, a new phenomenon that might have significant implications for our understanding of imprinting and future vaccine design. Furthermore, we show strong back-boosting in adults to an HA that carries the K133 epitope, and we provide evidence the IVPM binding data might serve as correlate of protection. While the current study is descriptive in nature and has limitations in terms of sample size, our findings are highly significant since they inform the design of future studies to elucidate imprinting effects and the longitudinal dynamics of antibody responses to influenza virus infection. These insights might open up new avenues for broadly protective of even universal influenza virus vaccine strategies.

## MATERIALS AND METHODS

### Participants and study procedures.

We performed a case-ascertained study to examine susceptibility to influenza virus infections in households in Managua, Nicaragua. Briefly, index cases of influenza and their household contacts were enrolled into the study and monitored closely for signs and symptoms of influenza virus infection. A nasal and oropharyngeal swab sample was collected at enrollment and every 2 to 3 days for up to 5 sequential respiratory samples per participant. A blood sample was collected at enrollment and 3 to 5 weeks later. All PCR^+^ subjects in this analysis were positive for H1N1. Influenza vaccination in the overall study population was very low, with just 10 out of 300 household contacts having received the influenza vaccine in that year. One vaccinated child was included in this analysis, and that child produced a narrow H1 response. There were multiple subclinical infections that occurred in the overall study; however, only one subclinical infection was included in this analysis set, a PCR^+^ adult that had a broad group 1 and group 2 HA response. Participation in the study was high, with nearly all houses invited to participate agreeing to participate; however, male adult participation was lower, as many were gone from their households from morning until night, and we were thus unable to contact them to invite them to participate in the study. Participants were excluded from this analysis if sufficient blood sample volume was not available. Ethical approval was obtained from the institutional review boards of the Ministry of Health, Nicaragua (CIRE 06/07/10-025) and the University of Michigan (HUM 00091392). Written informed consent was obtained from all adult participants, and proxy written informed consent was obtained for all children. Assent was obtained from children aged 6 and older.

### Recombinant proteins.

Recombinant HAs were produced using recombinant baculoviruses expressing soluble HAs with trimerization domains and hexahistidine tags. An Sf9 insect cell line (ATCC CRL-1711) was used to propagate the baculovirus, which was then used to infect BTI-*TN*-5B1-4 cells, for efficient secretion of recombinant HA. Recombinant HA was purified from cell supernatant using Ni-nitrilotriacetic acid resin columns. The procedure is described in detail in published protocols (35, 36).

### Influenza virus protein microarrays.

The IVPMs were generated and probed similarly to protocols described before (13). Briefly, arrays of recombinant HA were spotted on Nexterion E epoxysilane-coated glass slides (Schott, Mainz, Germany). Eight HAs were included in each array and spotted in triplicate, and 24 arrays were spotted on each slide. Each HA spot had a volume of 30 nl and was spotted at a concentration of 100 μg/ml in phosphate-buffered saline (PBS). After spotting, slides were incubated for 2 h at >95% relative humidity at room temperature and then allowed to dry. Slides were inserted into 96-well microarray gaskets (Arrayit, Sunnyvale, CA, USA) and blocked with 3% milk in PBS containing 0.1% Tween 20 (PBST) for 90 min. After the blocking solution was removed, sera were added at a starting concentration of 1:100 in 1% milk-PBST at a volume of 100 μl/array, and two 10-fold dilutions were performed across each slide. Sera were incubated on the slides for 1 h, and then the slides were washed three times with 220 μl/array PBST before the addition of 50 μl secondary antibody solution, composed of Cy3-labeled anti-human IgA secondary antibody and Cy5-labeled anti-human IgG secondary antibody diluted 1:400 and 1:1,500, respectively, in 1% milk-PBST. After 1 h, the secondary antibody solution was removed, and the arrays were washed three times with 220 μl/array PBST, removed from the 96-well microarray gaskets, rinsed with deionized water, and dried with an air compressor. Dried microarray slides were analyzed with a Vidia microarray scanner (InDevR, Boulder, CO, USA) at an exposure time of 1,000 ms. The AUC was calculated from median fluorescence as the total peak area above a fluorescence of 0.04. AUC values were adjusted based on the reactivity of a standard protein spotted in each array type, A/Perth/16/2009, using reactivity in array 1 as the standard. The AUCs of each array type were multiplied by the mean reactivity of A/Perth/16/2009 in array 1 divided by the mean reactivity of A/Perth/16/2009 in that array type.

IVPMs with monoclonal antibodies (MAbs) were performed as described above but with starting dilutions of 30 μg/ml MAb serially diluted in 1% milk-PBST 1:5 eight times and incubated with 100 μl/array Cy5-labeled anti-IgG secondary antibody diluted 1:3,000 in 1% milk-PBST.

### ELISA.

Recombinant HA proteins were coated onto 96-well Immulon 4 HBX plates (Thermo Scientific, Waltham, MA, USA) in coating solution (KPL) overnight at 4°C. The coating solution was removed, and each well was blocked with 220 μl/well of 3% nonfat milk in PBST for 2 to 3 h at room temperature. After removing the blocking solution, human sera were added at a starting concentration of 1:200 in 1% milk-PBST and serially diluted 1:2 10 times in 1% milk-PBST for final volumes of 100 μl/well and incubated for 1.5 h at room temperature. Sera were then removed, and plates were washed three times with 300 μl/well PBST. Fifty μl/well horseradish peroxidase (HRP)-labeled anti-human IgG secondary antibody, diluted 1:3,000 in 1% milk-PBST, was added to each well and incubated for 1 h at room temperature. Secondary antibody was then removed, the plates were washed four times with 300 μl/well PBST, and 100 μl/well SigmaFast OPD (*o*-phenylenediamine dihydrochloride [Sigma, St. Louis, MO, USA]) was added. After 10 minutes, 50 μl/well 3 M HCl was added, and the optical density of each well was measured at 490 nm using a Synergy H1 hybrid multimode microplate reader (BioTek, Winooski, VT, USA). The area under the curve (AUC) was calculated as the total peak above three standard deviations above the mean optical density of background wells, which were incubated with 1% milk-PBST instead of serum.

### Multidimensional scaling.

Three-dimensional antibody landscapes were generated to visualize the magnitude and breadth of serum reactivity to different HAs. The horizontal planes in the antibody landscapes were generated by assigning HAs *x*-*y* coordinates generated by multidimensional scaling of amino acid sequence differences between HAs. The sequence distance between strains was defined as the number of different amino acids between HAs in the multiple-sequence alignment of HAs included in the array. The Scaling by MAjorizing a COmplicated Function (SMACOF) algorithm was used to minimize the sum of squared errors between the Euclidean distance in the two-dimensional (2D) plane and the HA sequence distance. For each HA, an antibody landscape surface was generated from geometric mean AUC values using multilevel B-splines for pre- and postexposure sera (12).

### Statistical analysis and viral sequence analysis.

Statistical analysis was performed in GraphPad Prism 7.0 and R. Antigenic altitudes were compared using an unpaired *t* test, Pearson correlation analyses and linear regressions were used to compare IVPM and ELISA data and to compare IgA and IgG IVPM data, respectively, and AUC analyses were performed on IVPM and ELISA data. Logistic regression was used to measure the correlates of protection from preexisting antibodies. Crude models and models adjusted for age and sex were run; this was done using the “glm” function in R. Plots in Fig. 6 were created using ggplot2.

Sequences for K133 epitope analysis were collected from the Global Initiative for Sharing all Influenza Data (GISAID) from the years 1918 to 2019. They were then sorted by the year of isolation. Within each year, a subset of randomly selected sequences was compiled so that were 100 sequences or fewer if there were not 100 isolates for a specific collection year. Sequences for each year were aligned using MUSCLE. The residue at position 133 was then examined for the presence or absence of K133. The percent prevalence of K133 was calculated by the number of K133 residues in the yearly data set divided by the total isolates included for that year.
