# Supplementary material for: Influenza Virus Infection Induces a Narrow Antibody Response in Children but a Broad Recall Response in Adults
Source: mBio. 2020 Jan 21;11(1):e03243-19. doi: 10.1128/mBio.03243-19 (PMC6974575; doi:10.1128/mBio.03243-19)
Supplement: FIG S1 [file mBio.03243-19-sf001.docx]

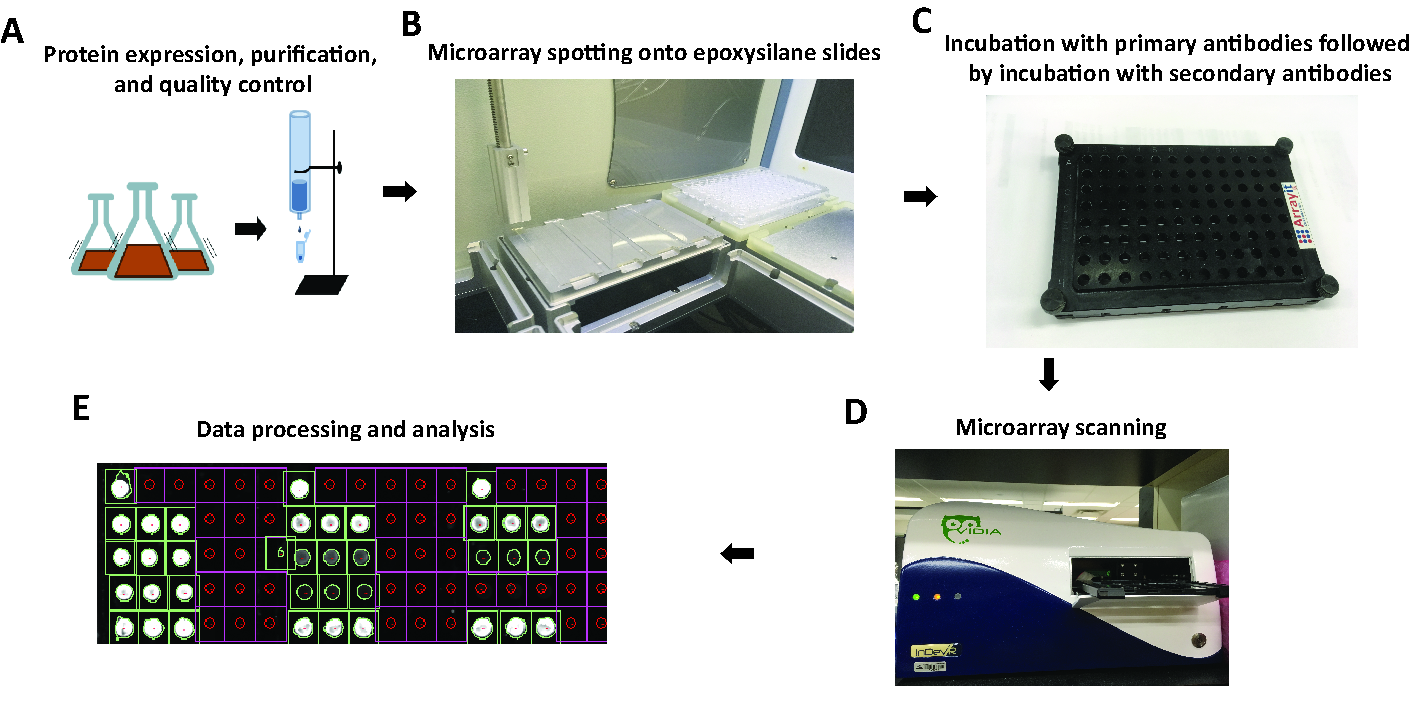


**Supplemental Figure 1. Schematic of the influenza virus protein microarray pipeline. A** Proteins are expressed in a baculovirus expression system, purified on Ni-nitrotriacetic acid resin columns and **B** spotted on epoxysilane-coated glass slides. **C** The slides are then probed with serum dilutions and fluorescently-labeled secondary antibodies in gaskets that house 4 glass slides per gasket. Each slide is further subdivided into 24 wells by the gaskets, in which serial dilutions are performed. **D** Slides are read on a microarray plate reader and **E** the data is analyzed.
