# Supplementary material for: Influenza Virus Infection Induces a Narrow Antibody Response in Children but a Broad Recall Response in Adults
Source: mBio. 2020 Jan 21;11(1):e03243-19. doi: 10.1128/mBio.03243-19 (PMC6974575; doi:10.1128/mBio.03243-19)
Supplement: FIG S2 [file mBio.03243-19-sf002.docx]

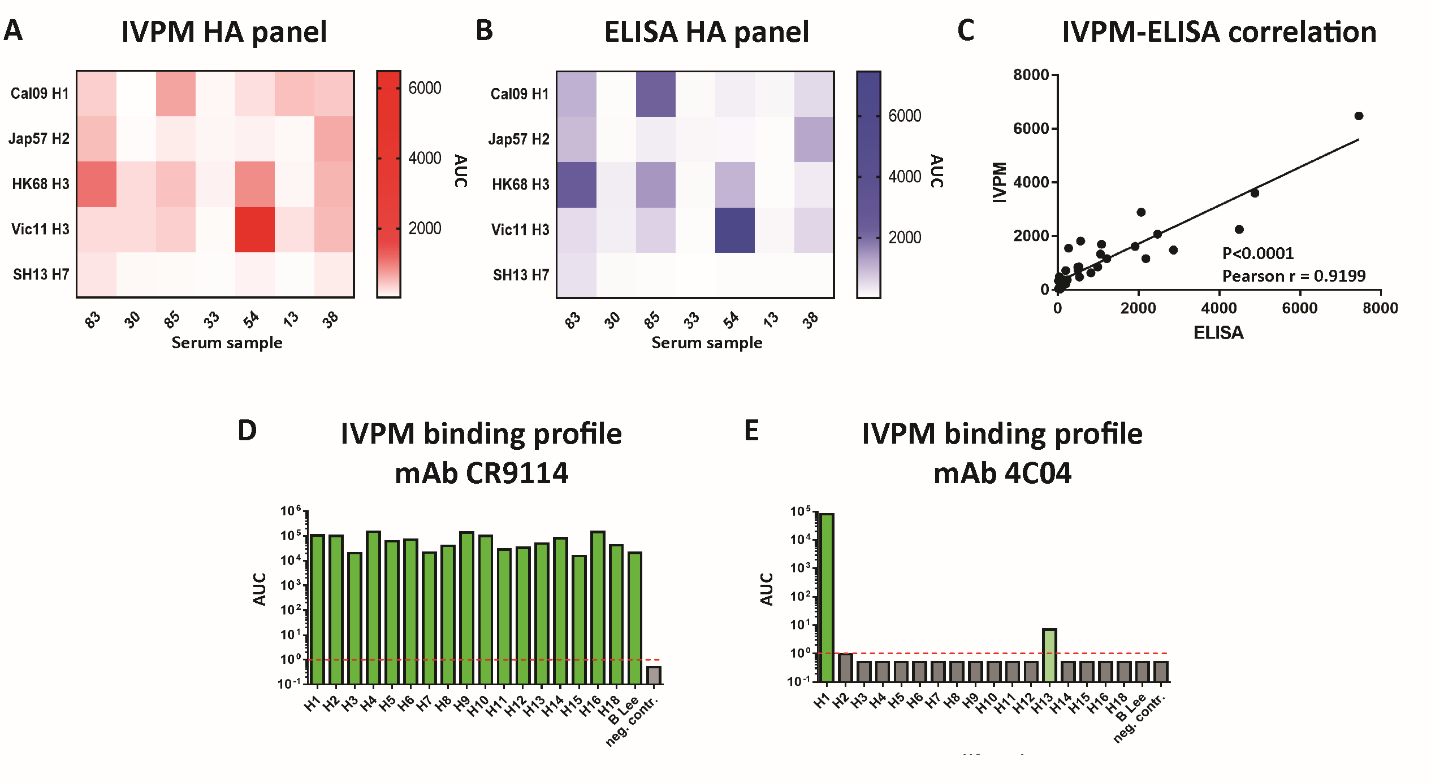


**Supplemental Figure 2. Titers measured with the IVPM technology correlate with ELISA titers. A** In a pilot study, sera from seven commercially obtained human serum samples were probed on the IVPM against five different recombinant HAs. The data is represented as a heatmap of AUC values. **B** shows the same serum/protein combinations but measured by ELISA. **C** shows correlation between IVPM and ELISA data. **D** shows mAb CR9114, which binds to all influenza A virus HA subtypes and influenza B virus HA in ELISA, also binds broadly to different HAs on the IVPM. **E** To demonstrate specificity, the IVPM was also probe with pandemic H1 specific (as assessed in ELISA) mAb 4C04. The mAbs shows the same specific pattern of reactivity on an IVPM.
